# Using Interaction between Cognitive and Motor Impairment for Risk Screening of Major Neurocognitive Disorders: Results of the EPIDOS Observational Cohort Study

**DOI:** 10.3390/brainsci12081021

**Published:** 2022-07-31

**Authors:** Olivier Beauchet, Jacqueline Matskiv, Yves Rolland, Anne-Marie Schott, Gilles Allali

**Affiliations:** 1 Departments of Medicine and Geriatrics, University of Montreal, Montreal, QC H3W 1W5, Canada; 2 Research Centre of the Geriatric University Institute of Montreal, Montreal, QC H3W 1W5, Canada; jacquelinematskiv@gmail.com; 3 Department of Medicine, Division of Geriatric Medicine, Sir Mortimer B. Davis Jewish General Hospital and Lady Davis Institute for Medical Research, McGill University, Montreal, QU H3T 1E2, Canada; 4 Lee Kong Chian School of Medicine, Nanyang Technological University, Singapore 636921, Singapore; 5 Department of Geriatric, Toulouse University Hospital, 31059 Toulouse, France; rolland.y@chu-toulouse.fr; 6 HESPER EA 7425, Hospices Civils de Lyon, Pôle de Santé Publique, Université Claude Bernard Lyon 1, 69229 Lyon, France; anne-marie.schott-pethelaz@chu-lyon.fr; 7 Leenaards Memory Center, Lausanne University Hospital and University of Lausanne, 1011 Lausanne, Switzerland; gilles.allali@chuv.ch

**Keywords:** older adults, epidemiology, cohort study, dementia, screening

## Abstract

Background and purpose: Cognitive and motor impairments are risk factors of major neurocognitive disorders (MNCD). Inability to name the date and use of a walking aid and/or history of falls are two items which are surrogate measures of cognitive and motor impairments. This study aims to examine the association of inability to name the date (i.e., cognitive impairment), use of a walking aid and/or history of falls (i.e., motor impairment) and their combination with incident MNCD in community-dwelling older adults. Methods: A total of 709 participants (mean age 79.8 ± 3.7; 100% female) of the EPIDémiologie de l’OStéoporose (EPIDOS) study recruited in Toulouse (France) were selected for this study. EPIDOS is an observational population-based cohort study with a 7-year follow-up period for Toulouse participants. Inability to name the date and use of a walking aid and/or history of falls were collected at baseline. Incident MNCD and their type (i.e., Alzheimer’s disease (AD) and non-AD) were diagnosed at the end of the 7-year follow-up. Results: Overall incidence of MNCD was 29.1%. Cox regressions revealed that inability to name the date and its combination with use of a walking aid and/or history of falls was associated with a significant increased incidence of MNCD (hazard ratio (HR) = 1.10 with *p* = 0.003 and HR = 1.81 with *p* = 0.011, respectively) and AD (HR =1.13 with *p* = 0.003 and HR = 2.80 with *p* = 0.016, respectively). Conclusions: Increased incident MNCD was reported when inability to name the date and use of a walking aid and/or history of falls were combined, suggesting that this combination of items may be used for risk screening of MNCD in the older population, especially for incident AD.

## 1. Introduction

Cognitive impairment is a typical risk factor of major neurocognitive disorders (MNCD) [1,2]. Motor impairment is also a risk factor for MNCD in the aging population [3,4,5,6,7]. For instance, mild parkinsonian signs—which are prevalent in aging—and slow walking speed have been associated with the occurrence of MNCD [5,6]. Both cognitive and motor impairments are independent risk factors of MNCD which may interact. Cognitive impairment is a risk factor of motor impairment and vice versa [1,3,4,5,6,7,8]. In addition, when they coexist, the risk of MNCD increases significantly [8,9,10]. The co-occurrence of slow walking speed and subjective cognitive complaint (SCC) in individuals free of MNCD defines motoric cognitive risk syndrome (MCR) [8]. MCR is a pre-MNCD stage and poses a greater risk of MNCD than each MCR component considered individually [8,9], suggesting a synergistic effect which may be used to screen MNCD in the older population [10].

Frailty is also associated with increased risk of MNCD [11]. Cognitive frailty is a clinical syndrome which combines physical and cognitive impairment [11,12]. Individuals with cognitive frailty have a higher risk of MNCD than those with physical frailty alone [11,12,13]. “Emergency Room Evaluation and Recommendations” (ER^2^) is a clinical tool which screens frailty and its related risk of adverse outcomes in older emergency department users [14]. Inability to name the date and use of a walking aid and/or history of falls are two ER^2^ items which are surrogate measures of cognitive and motor impairments. We hypothesized that, like the MCR components, interaction between these two ER^2^ items could be associated with an increased risk of MNCD. This study thus aims to examine the association of inability to name the date (i.e., cognitive impairment) and use of a walking aid and/or history of falls (i.e., motor impairment) and their combination with incident MNCD in community-dwelling older adults.

## 2. Material and Methods

### 2.1. Design

The “EPIDémiologie de l’OStéoporose” (EPIDOS) database was used for the present study [15]. EPIDOS is an observational population-based cohort study designed to examine risk factors for hip fracture in older French women. The participants selected for the present study were recruited in Toulouse (city in Southern France). They had an additional 3-year follow-up after the initial 4-year EPIDOS follow-up period, which included a final full cognitive assessment, which was performed at the University Hospital of Toulouse or at the participant’s home.

### 2.2. Population

The initial set of EPIDOS participants was composed of 7598 women, aged 75 and over, living in communities in five French cities (Amiens, Lyon, Montpellier, Paris and Toulouse). A total of 1462 (19.2%) participants were recruited in Toulouse. We excluded from this subset of participants those with a suspicion of MNCD at baseline using the threshold value of ≥3 incorrect answers on the Short Portable Mental Status Questionnaire (SPMSQ), and those without information on their cognitive status (i.e., no MNCD versus MNCD and its etiology coded as Alzheimer’s Disease (AD) vs. non-AD) at the end of follow-up [16]. A total of 709 (48.5% of the Toulouse EPIDOS participants) participants were finally selected for the present study.

### 2.3. Baseline Assessment

Age, living in residence, high education level (i.e., high school level or higher completed), living alone, frequency of contact with someone over the past week, number of drugs taken daily, measured weight (in kg) and height (in cm), regular physical activity (i.e., ≥ one hour a week during the past month), use of a walking aid, history of falls in the past 6 months and inability to name the date were recorded at baseline assessment using a standardized face-to-face physical examination. Age was stratified into two groups using the threshold value ≥ 85. Body mass index (BMI) was calculated. Overweight and/or obesity were defined as a BMI ≥ 25 kg/m^2^. Polypharmacy was defined as ≥5 drugs taken daily. Social isolation was defined as living alone and no contact with someone over the past week.

### 2.4. Definition of MNCD

At the end of the 7-year follow-up, a face-to-face cognitive assessment including the SPMSQ [10], the Mini Mental State Examination [17] and the Grober and Buschke test (i.e., Free and Cued Selective Reminding Test) was performed [18]. Data collected were analyzed by a geriatrician and a neurologist in a double-blind manner to determine the cognitive status of participants. DSM-IV criteria were used for the diagnosis of MNCD [13,14]. AD diagnosis was made using the criteria of the NINCDS-ADRDA Work Group [19,20,21,22]. Participants who satisfied DSM-IV criteria but not NINCDS-ADRDA criteria were classified with a diagnosis of non-AD. Participants were separated in four groups: no MNCD, all categories of MNCD, AD and non-AD.

### 2.5. Standard Protocol Approval and Patient Consents

The Research Ethics Boards (REB) of Toulouse University Hospital approved the EPIDOS protocol (protocol code EPIODS (@ and 1992/01/05). Written informed consent for research was obtained for all recruited EPIDOS participants.

## 3. Statistics

The participants’ baseline characteristics were described using means, standard deviation (SD), percentages and confidence intervals. Cox regressions were performed to examine the association of inability to name the date, use of a walking aid and/or history of falls and their combination (independent variables; separated model for each variable) with incident MNCD (dependent variable; separated model for each type of MNCD). All models are adjusted by age, place of living, education level, abnormal body mass index (i.e., ≥25 kg/m^2^), regular physical activity, polypharmacy and social isolation; *p*-values < 0.05 were considered statistically significant. All statistics were performed using SPSS (version 28.0; SPSS, Inc., Chicago, IL, USA).

## 4. Results

Table 1 shows the baseline characteristics of participants. The incidence of MNCD was 29.1%. AD was more incident than non-AD (15.5% vs. 13.5%). Cox regressions showed that inability to name the date and its combination with use of a walking aid and/or history of falls were significantly associated with an increased incidence of MNCD (hazard ratio (HR) = 1.10 with *p* = 0.003 and HR = 1.81 with *p* = 0.011) and AD (HR =1.13 with *p* = 0.003 and HR = 2.80 with *p* = 0.016) (Table 2). No significant association was found with incident non-AD. Use of a walking aid and/or history of falls was not associated with incident MNCD, including its subtypes.

## 5. Discussion

The findings show that an increased incidence of MNCD and AD were associated with inability to name the date alone and its combination with use of a walking aid and/or history of falls in EPIDOS participants. The greatest incidence of MNCD was reported when inability to name the date was combined with use of a walking aid and/or history of falls and AD.

We found that inability to name the date, but not use of a walking aid and/or history of falls, was associated with incident MNCD and AD. This result is consistent with a previous study which examined the association of MCR and its components (i.e., slow walking speed and SCC) with incident MNCD [6]. In this former study, the cognitive component of MCR (i.e., subjective cognitive complaint) and not slow walking speed was associated with incident MNCD in older community dwellers. An explanation of this specific association may be due to the fact that the onset of MNCD is characterized by cognitive impairment [1]. Inability to name the date may be assimilated as an objective cognitive impairment. In our study, selected participants were free of MNCD at baseline and only those with temporal impairment had significant risk of incident MNCD. Although specific motor impairment related to gait disorders or mild parkinsonian signs may predict cognitive decline, the association between cognitive impairment and incident MNCD is stronger than between motor impairment and incident MNCD [7,8,9]. Finally, there are more mixed results regarding the association between motor impairment and incident MNCD compared to cognitive impairment [7,8,9,10].

Our findings also revealed that the association between both items (i.e., inability to name the date and use of a walking aid and/or history of falls) and incident MNCD was significant and greater when combined, compared to the inability to name the date by itself. Again, this result is consistent with previous results reported on MCR [9,10]. Indeed, we report that the magnitude of this risk is two-fold when compared to the cognitive impairment item alone. This result highlights an interaction between cognitive impairment and motor impairment, which may be used to screen individuals at risk of MNCD and AD. Interestingly, inability to name the date alone or combined with use of a walking aid and/or history of falls did not predict non-AD. This contrast with the prediction of AD may be related to the heterogeneity of the patients included in the non-AD dementia group. Indeed, vascular dementia, Lewy bodies dementia, or other neurodegenerative conditions affecting the oldest old (i.e., PART, LATE) rely on different neuropathogenic mechanisms.

The 7-year duration of the prospective, observational follow-up and the sample size are the main strengths of the present study, but some limitations emerged. First, even if EPIDOS’s design was appropriate for the objective of our study, examining an association between the ER^2^ items and incident MNCD was not initially planned. Second, we selected only EPIDOS participants recruited in Toulouse, only about half of which were included in the present study, which may have introduced selection bias and impacted outcomes. Third, Cox models were adjusted for participants’ baseline characteristics, but residual confounders may still be present and modify the association between the ER^2^ items and incident MNCD. For instance, chronic morbidities may influence both cognitive and motor impairments, and thus their association with incident dementia [11]. We tried to control for the effects of comorbidities by adjusting for polypharmacy, which is a surrogate measure of accumulation of morbidities [23]. Finally, the generalization of the study findings does not apply to males, as EPIDOS included only women.

## 6. Conclusions

Increased incident MNCD was reported when inability to name the date and use of a walking aid and/or history of falls were combined, suggesting that these items may be used for risk screening of MNCD in the older population. Both items are easy to collect at the level of an older population, which creates new opportunities for MNCD risk identification and the preventive care of its modifiable risk factors [24,25].

## Figures and Tables

**Table 1:** Participants’ baseline characteristics and incident major neurocognitive disorders (n = 709).

Characteristics	Value	[95% CI]
Age (year)		
Mean ± SD	79.8 ± 3.7	[79.5; 80.1]
Age ≥ 85, n (%)	69 (9.7)	[7.5; 11.9]
Living in residence, n (%)	77 (10.9)	[8.6; 13.3]
Social isolation *, n (%)	276 (38.9)	[35.2; 42.4]
High education level ^†^, n (%)	299 (42.2)	[38.4; 45.7]
Number of drugs taken daily		
Mean ± SD	5.0 ± 2.9	[4.8; 5.3]
Polypharmacy ^‡^	388 (54.7)	[51.0; 58.4]
Body mass index (kg/m^2^)		
Mean ± SD	25.0 ± 3.9	[24.7; 25.3]
Overweight/Obese ^¶^	324 (45.7)	[42.01; 49.3]
Regular physical activity ^#^	297 (41.9)	[38.5; 45.8]
Use of walking aid and/or history of fall in the past 6 months	227 (32.0)	[29.5; 34.6]
Inability to name day’s date	147 (20.7)	[18.1; 23.7]
Incident major neurocognitive disorders, n (%)		
All categories	206 (29.1)	[25.5; 32.2]
Non-Alzheimer’s disease	96 (13.5)	[11.0; 16.0]
Alzheimer’s disease	110 (15.5)	[12.7; 18.0]

SD: standard deviation; CI: confidence interval; *: living alone and no contact with someone over the past week; †: high school and greater; ‡: number of drugs taken daily > 5; ¶: value ≥ 25 kg/m^2^; #: at least one recreational physical (walking, gymnastics, cycling, swimming or gardening) activity for at least one hour a week for the past month or more.

**Table 2:** Cox regressions showing the association of inability to name day’s date, use of walking aid and/or history of falls and their combination (independent variable; separated model for each variable) and incident major neurocognitive disorders (all categories, non-Alzheimer’s disease, Alzheimer’s disease; dependent variable; separated model for each category) in EPIDOS participants (*n* = 709).

	Major Neurocognitive Disorders								
	All Categories			Non-Alzheimer’s Disease			Alzheimer’s Disease		
	HR	[95% CI]	*p*-Value	HR	[95% CI]	*p*-Value	HR	[95% CI]	*p*-Value
Inability to name the day’s date	1.10	[1.03; 1.17]	**0.003**	1.06	[0.97; 1.16]	0.226	1.13	[1.05; 1.23]	**0.003**
Use of walking aid and/or history of falls	1.01	[0.96; 1.08]	0.646	0.98	[0.90; 1.07]	0.679	1.04	[0.96; 1.13]	0.316
Inability to name the day’s date AND use of walking aid and/or history of falls	1.81	[1.15; 2.85]	**0.011**	1.53	[0.76; 3.09]	0.235	2.08	[1.14; 3.78]	**0.016**

HR: hazard ratio; CI: confidence interval; all models are adjusted by age, place of living, social isolation, education level, body mass index ≥ 25 kg/m^2^, regular physical activity and polypharmacy; *p*-value significance (i.e., <0.05) indicated in bold.

## Data Availability

Access to the EPIDOS Database can be obtained by contacting Olivier Beauchet via Olivier.beauchet@umontreal.ca.
